# The association between Myers-Briggs Type Indicator and Psychiatry as the specialty choice

**DOI:** 10.5116/ijme.5698.e2cd

**Published:** 2016-02-06

**Authors:** Chong Yang, George Richard, Martin Durkin

**Affiliations:** 1 Department of Neuropsychiatry and Behavioral Science, University of South Carolina School of Medicine, Columbia, South Carolina, USA; 2 Careers in Medicine Program, Association of American Medical Colleges, Washington, District of Columbia, USA; 3 Research Compliance Department, Palmetto Health, Columbia, South Carolina, USA

**Keywords:** Personality, Myers-Briggs Type Indicator, MBTI, Psychiatry, medical students

## Abstract

**Objectives:**

The purpose of this pilot study is to examine the association between Myers-Briggs Type Indicator (MBTI) and prospective psychiatry residents.

**Methods:**

Forty-six American medical schools were contacted and asked to participate in this study. Data were collected and an aggregated list was compiled that included the following information: date of MBTI administration, academic year, MBTI form/version, residency match information and student demographic information. The data includes 835 American medical students who completed the MBTI survey and matched into a residency training program in the United States. All analyses were performed using R 3.1.2.

**Results:**

The probability of an introvert matching to a psychiatry residency is no different than that of an extravert (p= 0.30). The probability of an intuitive individual matching to a psychiatry residency is no different than that of a sensing type (p=0.20). The probability of a feeling type matching to a psychiatry residency is no different than that of a thinking type (p= 0.50). The probability of a perceiving type matching to a psychiatry residency is no different than that of a judging type (p= 0.60).

**Conclusions:**

Further analyses may elicit more accurate information regarding the personality profile of prospective psychiatry residents. The improvement in communication, team dynamics, mentor-mentee relationships and reduction in workplace conflicts are possible with the awareness of MBTI personality profiles.

## Introduction

The Myers-Briggs Type Indicator (MBTI) is a psychometric test developed in the 1940s by Isabel Myers and her mother Katherine Briggs.^1^ Based on the theory of psychological type by Carl G. Jung, the MBTI is a useful tool that helps individuals identify their personality preferences. It offers an explanation of the individual’s decision-making process, perception of the world and mechanism of interacting with the external environment.

The current version of the MBTI form M consists of 93 questions and requires 15-25 minutes to complete the questionnaire. The results are presented through four dichotomies: Extraversion vs Introversion, Sensing vs Intuition, Thinking vs Feeling and Judging vs Perceiving. These four dimensions lead to 16 different possible combinations, or types. Since the first publication in 1962, the MBTI tool has been administered to millions of people worldwide as a tool for team building, leadership and coaching, conflict management and career development.^1^

Medical students, resident physicians and attendings have all been surveyed with the MBTI in the past. Myers and McCaulley surveyed medical students with the MBTI during the 1960s and 1970s.^2^ Stilwell et al. explored additional questions with data collected from the 1980s and 1990s.^2^ Stilwell explored differences in MBTI profiles of current medical students (comparing their data to data collected from the 1970s), identified differences between MBTI profiles of men and women and explored associations between particular types and medical career choices. Individuals with a personality preference of Introversion and Feeling were more likely to choose primary care specialties. Individuals with a personality preference of Extraversion and Thinking are more likely to choose surgical specialties. Stilwell also identified a trend towards more Judging types in the medical profession.

Markert et al. also examined the association between personality characteristics and specialty choice.^3^ Markert administered the Neuroticism-Extraversion-Openness Personality Inventory Revised (NEO PI-R) to four Tulane University School of Medicine classes. One difference among the specialties was the higher openness scores for graduates entering psychiatry. Markert defined openness as the focus on intellectual curiosity and independent judgment (example facets: fantasy, aesthetics, and feelings).

Others have focused on the MBTI profiles of a specific medical specialty.^4^^,^^5^^,^^6^^,^^7^ Zardouz et al. identified Introversion, Sensing, Thinking and Judging as the most prevalent personality traits in prospective otolargyngology applicants.^4^ Swanson et al. reported Introversion, Sensing, Thinking and Judging as the most common personality type in surgery residents.^5^ Boyd and Brown identified Extraversion, Intuition, Thinking and Judging to be the most common personality profile of Emergency Department medical staff.^6^ However, there are no studies examining the MBTI profile of prospective psychiatry residents in the medical literature.

The aim of this pilot study is to examine the association between MBTI and prospective psychiatry residents. In the past, published research identified the MBTI profiles of different medical specialties.^4^^,^^5^^,^^6^^,^^7^ However, the data are outdated with most recent data collected prior to the year 2000 and therefore it may not reflect the current medical school population.

There are significant changes in both medical schools and residency programs since the year 2000 that make this research worthwhile. The strong initiative to increase the number of medical schools and medical school graduates in the United States may change the culture of residency programs. Another factor to take into consideration is the Accreditation Council for Graduate Medical Education (ACGME) new work hour restrictions and new supervision standards that went into effect July 2011.^8^ Swanson et al. proposed that as residency training programs continue to evolve their curriculum to satisfy ACGME standards, the personality profiles of residents could change as well. A change was evident in their study as Introversion, Sensing, Thinking and Judging was determined to be the most common personality profile of surgeons. Previous studies identified Extraversion, Sensing, Thinking and Judging as the most common personality type of surgeons.^5^

Previous studies examined numerous variables in the medical student’s decision to select a specialty.^9^^-^^12^ Vaidya et al. identified the relative influence of temperament and character in association with specialty choice.^9^ Sierles identified academic rank of the medical student’s training director in psychiatry as the strongest predictor of the medical student’s interest in selecting psychiatry as the career choice.^10^ Gowans et al. described the early interest in psychiatry as the strongest predictor for whether a student chooses psychiatry as a career.^11^ Education debt is also another variable that has been examined in association with specialty choice.^12^ However, the association between Myers-Briggs Type Indicator and Psychiatry as the specialty choice has not been examined to our knowledge.

The authors suspect that aspiring psychiatrists have the tendency to favor the MBTI combination of Introversion, Intuition, Feeling and Judging. The reserved and independent nature of introverts may provide additional comfort for patients who disclose confidential information in the conventional, private 1 to 1 setting. Intuition is predicted as psychiatrists attempt to process information using more abstract information and subjective measures compared to their other colleagues in medicine. Thinking-Feeling is a function of decision-making as ‘thinkers’ prefer to make decisions with logic and reason while ‘feelers’ prefer to make decision based on empathy. As empathy is a hallmark attribute for psychiatrist, the authors predict a higher tendency for Feeling among prospective psychiatry residents. The assumption for Judging over Perceiving is based on previous studies regarding MBTI and different specialties in medicine.^2^^,^^4^^,^^5^^,^^6^ The preference for organization and plan is a shared quality among all medical students and physicians.

The identification of personality types in medical students who pursue psychiatry residency training has its merits. The findings may serve an additional resource for medical students who are interested in psychiatry and also for psychiatry program directors as they educate their psychiatry interns according to their strengths. Educational objectives and curricular development are future considerations that may benefit from this study. The improvement in communication, team dynamics, mentor-mentee relationships and reduction in workplace conflicts are possible with the awareness of MBTI personality profiles.

## Methods

### Participants

In 2011, the Association of American Medical Colleges (AAMC) surveyed all M.D. medical schools in the US to determine if any personality measures were administered to their medical students. Sixty-three percent of the schools responded to the survey and 61% of these medical schools (N=46) reported using the MBTI. These forty-six medical schools were contacted and asked to participate in this study.

### Ethical approval

The research project is not considered to be human subjects research. This research was considered to be “Not Human Subjects Research” per IRB Pro00031551 on 2/20/2014. The study has been approved through the PHARR process at Palmetto Health on 2/21/2014.

### Data collection

We collected data between January 1-May 31, 2014 and an aggregated list was compiled that included the following de-identified information: date of MBTI administration, academic year, MBTI form/version, residency match information (medical specialty, type of residency training program – academic or community-based) and student demographic information (sex, age, race/ethnicity). American medical schools accredited by the Liaison Committee on Medical Education that administered the MBTI from 2000-present and had data available for use were encouraged to participate in the study.

### Data analyses

All analyses were performed using R 3.1.2 (R Foundation for Statistical Computing, Vienna, Austria).^13^ The data did not contain any variables which allows for the identification of the medical students, their medical schools or the residency training programs to which they had been accepted.

The authors originally made the assumption that 23 of the 46 medical schools would agree to participate, with an average of 120 students per school matching per year. It was assumed that 5% would match to psychiatry, giving a total of 27,600 observations (1,380 of whom match to psychiatry and 26,220 of whom did not match to psychiatry-most of whom did not apply to psychiatry programs). As the response rate was lower than expected and this is the first study to investigate the association between personality factors and being accepted into a psychiatry residency program, the analyses were more exploratory and descriptive in nature.

## Results

The authors collected MBTI data from four medical schools in the United States: Virginia Commonwealth University School of Medicine, University of Illinois College of Medicine at Peoria, University of Nevada School of Medicine and University of South Carolina School of Medicine. The data includes 835 medical students who completed the survey and matched into a residency program. Twenty eight (28) of the 835 medical students matched into a psychiatry residency program. The most common personality types among prospective psychiatry residents include Introversion, Intuition and Judging personality traits (Table 1). There was no difference between Thinking and Feeling among aspiring psychiatrists.

**Figure 1:**
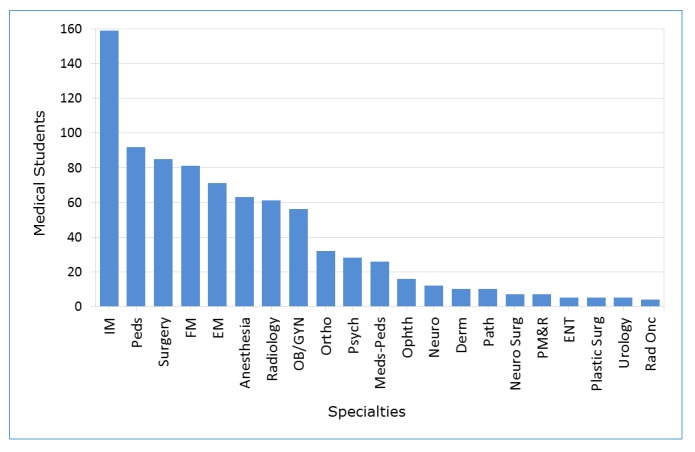
Match data for all specialties

The authors also collected MBTI data for medical students who matched into other specialties. These specialties include internal medicine, pediatrics, general surgery, family medicine and others (Figure 1). The most prevalent personality traits among all other medical students include Extraversion, Sensing, Thinking and Judging. However, there was no statistically significant association between any of the four dichotomies and the preference for psychiatry over other specialties. The probability of an introvert matching to a psychiatry residency is no different than that of an extravert (p=0.30). The probability of an intuitive individual matching to a psychiatry residency is no different than that of a sensing type (p=0.20). The probability of a feeling type matching to a psychiatry residency is no different than that of a thinking type (p=0.50). The probability of a perceiving type matching to a psychiatry residency is no different than that of a judging type (p=0.60).

**Table 1:** MBTI profile comparing counts (percentages) of medical students who matched into psychiatry residency programs versus other residency programs

Personality preference	Psychiatry (n=28)		Other Specialties (n=807)	
	Count	Percent (95% CI)	Count	Percent (95% CI)
Introversion	16	57.1% (37.2, 75.5)	381	47.2% (43.7, 50.7)
Extraversion	12	42.9% (24.5, 62.8)	426	52.8% (49.3, 56.3)
RR^*^ = 1.47 (0.70, 3.07)				
Intuition	16	57.1% (37.2, 75.5)	363	45.0% (41.5, 48.5)
Sensing	12	42.9% (24.5, 62.8)	444	55.0% (51.5, 58.5)
RR = 1.60 (0.77, 3.35)				
Feeling	14	50.0% (30.6, 69.4)	352	43.6% (40.2, 47.1)
Thinking	14	50.0% (30.6, 69.4)	455	56.4% (52.9, 59.8)
RR = 1.28 (0.62, 2.65)				
Perceiving	13	46.4% (27.5, 66.1)	335	41.5% (38.1, 45.0)
Judging	15	53.6% (33.9, 72.5)	472	58.5% (55.0, 61.9)
RR = 1.21 (0.58, 2.52)				

*RR = relative risk with 95% confidence interval. P-value is for the Χ^2^ test for the null hypothesis that the relative risk is 1.

## Discussion

This pilot study examines the personality profile of medical students who matched into a psychiatry residency program. The majority of students exhibited the profile of Introversion over Extraversion, Intuition over Sensing, Judging over Perceiving and no preference for Thinking vs Feeling. However, the results are not statistically significant and future studies are necessary to identify any association between MBTI and the selection of psychiatry as a specialty.

There are several limitations to this study that are noteworthy. First, not all medical schools had the opportunity to participate in this study. Forty-six medical schools were contacted due to their response from a previous survey and only 4 medical schools participated in this study. Another limitation is the small sample size of 28 medical students who matched into psychiatry residency training programs. The small sample size may not be an accurate reflection of general psychiatry applicants and limits the ability to extrapolate the results to general psychiatry applicants. Due to the paucity of participants and for the protection and prevention of identifying the participating medical students, demographic information including sex, age and race/ethnicity were not examined in detail.

We also accepted all forms of MBTI and medical students at different points in their medical education to increase the sample size. Both MBTI Form M and Form G were included in this study. Form M is the updated and current version. Form M has higher test-retest consistency and reliability compared to the previously administered Form G.^14^ While the majority of the data represent medical students in their first and second years of medical education, data from third year and fourth year medical students were also included. There is a possibility that these variables may have influenced the study.

It is also important to note that this study was conducted to determine the association between MBTI and prospective psychiatry residents. Students should not be discouraged from applying to psychiatry regardless of their MBTI profile. Mowbray et al. reported numerous factors that influence the choice of career.^15^ The authors agree that it is inappropriate to focus on a single factor such as MBTI to select a specialty.

## Conclusions

In summary, this pilot study examined the association between MBTI and prospective psychiatry residents. Aspiring psychiatrists showed preferences of Introversion over Extraversion, Intuition over Sensing, Judging over Perceiving and no preference for Thinking vs Feeling. Due to the small sample size, it is difficult to extrapolate the findings. Further analyses may elicit more accurate information regarding the personality profile of prospective psychiatry residents.

### Conflict of Interest

The authors declare that they have no conflict of interest.
